# Knowledge and Attitude of Saudi Women Towards Postmenopausal Symptoms and Management: A Cross-Sectional Study From Qassim Region, Saudi Arabia

**DOI:** 10.7759/cureus.106096

**Published:** 2026-03-29

**Authors:** Renad A Almugbel, Rand Albahli, Amel A Sulaiman

**Affiliations:** 1 Family Medicine, Family Medicine Academy, Qassim Health Cluster, Buraidah, SAU; 2 Family Medicine, Family Medicine Academy, Qassim Health Cluster, Burdaiah, SAU; 3 Preventive Medicine, Family Medicine Academy, Qassim Health Cluster, Buraidah, SAU

**Keywords:** clinical symptoms, cross sectional studies, knowledge, postmenopause, saudi women

## Abstract

Background: Post menopause marks a significant transition in a woman's life. It is often accompanied by various symptoms that can impact her physical health, emotions, and social interactions. This study aims to assess the knowledge and attitude of Saudi women towards postmenopausal symptoms and their management.

Methods: A cross-sectional study was conducted among Saudi women attending primary healthcare centers (PHCCs) in the Qassim region, Saudi Arabia. A convenience sampling method was used, and a pretested, self-administered online questionnaire was administered to women during PHCC visits. The instrument assessed sociodemographic characteristics, postmenopausal symptoms severity, and knowledge regarding postmenopausal symptoms and management. Data were analyzed using Statistical Package for Social Sciences (SPSS).

Results: Among the 401 recruited Saudi women, 41.6% (n=167) were aged 46-55 years, and most were university graduates, 58.9% (n=236). About 48.1% (n=193) were considered to have severe postmenopausal symptoms. 38.4% (n=154) of participating women had moderate knowledge of the postmenopausal symptoms and management. Increased knowledge was associated with older age, higher monthly income, and more children. Interestingly, we found that the knowledge score was positively correlated with the Menopause Rating Scale (MRS) total score (r=0.261; p < 0.001).

Conclusion: Women in the Qassim region of Saudi Arabia tend to have a basic understanding of menopause. However, there are important gaps in recognizing symptoms and discussing them with healthcare providers. Knowledge of menopause varies significantly by factors such as age, income, and number of children. This highlights a significant need for personalized educational efforts aimed at empowering women to make informed decisions about their menopausal care.

## Introduction

Menopause is a natural and inevitable biological process that marks the permanent end of menstruation, typically occurring between the ages of 45 and 55 as a result of the cessation of ovarian function [1,2]. The menopause experience varies from woman to woman and is influenced by factors such as health, age, and cultural background [1].

A key concern for postmenopausal women is the onset of various symptoms, with vasomotor disturbances like hot flashes and night sweats affecting up to 75% of women [3,4]. These symptoms are often the most bothersome and are commonly the reason women seek medical advice [3,4]. In addition, urogenital issues such as vaginal dryness and urinary urgency are prevalent and often effectively managed through estrogen therapy [2,3].

Postmenopausal women may also face musculoskeletal challenges, including back pain and an increased risk of fractures due to osteoporosis. Hormone therapy (HT) has been shown to be beneficial in preventing these issues [3]. Mental and emotional symptoms, including mood swings, irritability, and insomnia, are also significant concerns, with sleep disturbances often worsening cognitive and psychological outcomes [3,5].

In Saudi Arabia, studies have highlighted the frequency of muscle and joint pain, physical exhaustion, heart discomfort, and hot flashes among postmenopausal women, with psychological distress also being reported in a substantial number of cases [6,7]. However, awareness of menopause's full impact remains limited, with many women underestimating the associated physical and psychological effects [7]. 

Despite the documented prevalence of menopausal symptoms in Saudi Arabia, there is still a gap in understanding how specific regional populations, such as those in the Qassim region, perceive and manage this transition. Therefore, this study aimed to evaluate the knowledge about postmenopausal symptoms among Saudi women attending primary healthcare centers in Buraidah city, Qassim region, Saudi Arabia. Specifically, the research sought to assess the participants' knowledge and attitudes toward menopause and its management. Additionally, the study aimed to identify the sociodemographic and clinical factors that influence these knowledge levels and attitudes, providing essential data to guide targeted educational interventions and improve the quality of life for women in the region.

## Materials and methods

Study design

This descriptive, cross-sectional, facility-based study was conducted between October 2024 and October 2025 to assess the knowledge and attitudes of Saudi women regarding postmenopausal symptoms and management. This design was selected for its effectiveness in capturing data at a specific point in time, making it ideal for analyzing current awareness levels and perceptions. Furthermore, it facilitated the examination of potential associations between demographic factors and knowledge or attitudes regarding postmenopausal symptoms and their management.

Study area 

The research was conducted among Saudi women attending the primary healthcare centers (PHCCs) in Buraidah city, Qassim region, Saudi Arabia. Buraidah is the capital of the Al-Qassim region and accounts for approximately 60% of the region's total population. The city is supported by 43 PHCCs. These facilities are part of a broader network of 156 centers across Al-Qassim, which serve a total population of 1,336,179 consisting of 926,490 Saudi citizens and 409,689 non-Saudi residents [8,9]. Beyond its health infrastructure, the region is a vital agricultural center, with Buraidah serving as a primary driver of the Kingdom's renowned date production industry. 

Study population and sample size

The study population consisted exclusively of female attendees in primary healthcare settings in Buraidah city, Qassim region of Saudi Arabia. The eligibility criteria included women living in the Qassim region who demonstrated a willingness to participate; those who were non-Saudi residents and those with severe cognitive impairment or an inability to communicate were excluded from recruitment. The sample size was determined using the OpenEpi sample size calculator. According to the General Authority for Statistics (2023), the total population of the Qassim region is 1,336,179 [9]. Assuming a 50% prevalence of menopausal symptoms in women, our goal was to achieve a 95% confidence level with a 5% margin of error. The calculated sample size was 383 women. To account for potential missing data and ensure robustness, the sample size was increased by 10%, bringing the total to 421 female participants.

Sampling technique

The study employed a nonprobability convenience sampling method, involving the selection of female primary healthcare attendees who were readily available and willing to participate. Investigators approached women in the waiting areas of the selected PHCCs to invite their participation. This method was particularly advantageous for accessing the target population in the Qassim region, allowing for efficient data collection while aiming for a diverse representation of women attending PHCCs in Buraidah city. To minimize selection bias, data collection was conducted at varying times of the day, including both morning and evening sessions, throughout the study period.

Data collection technique and tool

Data were collected using a structured questionnaire administered by the investigators to participants at PHCCs in Buraidah city. The questionnaire was designed by the authors after an extensive literature review of previous studies [6,7,10,11]. To ensure content validity, the items were aligned with established clinical indicators of menopause and reviewed by the research team for relevance. The questionnaire was pretested for face validity to ensure the wording was clear and unambiguous. The data collectors were thoroughly trained to administer the questionnaire, ensuring consistent and accurate data collection.

Questionnaire Structure

The questionnaire consisted of two main sections. First, sociodemographic information was gathered, including age, education level, occupation, residence, marital status, parity, monthly income, and menopausal status. Second, a knowledge and attitudes assessment evaluated participants' understanding of menopause and its associated symptoms using the Menopause Rating Scale (MRS) and explored perceptions and behaviors regarding menopause and its management [11]. The complete, two-section questionnaire is available in Appendix 1. 

Pilot Study

Before the main data collection, a pilot study was carried out with 10 participants to assess the instrument's face and content validity, specifically focusing on clarity, comprehensibility, and cultural relevance. This pilot phase used cognitive debriefing to identify and improve ambiguous items. Although the pilot data were excluded from the final analysis, specific adjustments were made to the Arabic translation of the MRS hot flush descriptors and the questions related to irritability to enhance the tool's effectiveness within the local Qassim context.

Questionnaire criteria

Postmenopausal symptoms have been evaluated using the MRS, a validated tool for women to gauge symptom severity and its impact on overall health and quality of life. The scale consists of 11 items that reflect common symptoms, including hot flushes, heart discomfort, trouble sleeping, joint and muscle pain, depression, irritability, anxiety, fatigue, sexual problems, bladder issues, and vaginal dryness. Women are asked to rate each symptom on a five-point scale, where 0 means no symptoms, and 4 indicates very severe symptoms. Scores range from 0 to 44, with higher scores indicating greater symptom severity. MRS has been shown to have good internal consistency (Cronbach's α > 0.7) and construct validity across diverse populations and is available in multiple validated language versions to ensure cross-cultural applicability [11,12]. The MRS is reliable across various groups and available in multiple languages, making it accessible to women everywhere. Healthcare providers typically administer it at the start and during follow-ups to monitor progress [13]. The total MRS score was classified into four categories to determine symptom severity: No/Little (score 0-4), mild (score 5-8), moderate (score 9-16), and severe (score 17+) [14].

The knowledge of Saudi women regarding postmenopausal symptoms and management was assessed using a 26-item questionnaire, with correct answers coded as '1' and incorrect as '0'. Total knowledge scores ranged from 0 to 26, where higher scores indicated greater knowledge. The level of knowledge was categorized using the 33rd and 66th percentiles as cutoff points. Based on the distribution of scores, participants were classified into three levels: poor knowledge (scores 0-12; < 33rd percentile), moderate knowledge (scores 13-15; 33rd-66th percentile), and good knowledge (scores 16-26; > 66th percentile)[15].

Statistical analysis

All categorical variables were presented using numbers and percentages, while all continuous variables were summarized using mean ± SD. The differences in knowledge scores across sociodemographic characteristics of Saudi women were examined using the Mann-Whitney U test and the Kruskal-Wallis H test. The Spearman correlation coefficient was also used to assess the relationship between knowledge and MRS scores. Statistical normality was measured using the Kolmogorov-Smirnov test. Based on the plot, both knowledge and MRS scores follow a non-normal distribution. Therefore, the non-parametric tests were applied. All statistical analyses were performed using the Statistical Package for Social Sciences (SPSS), version 26.0 (IBM Corp., USA)

## Results

**Figure 1:**
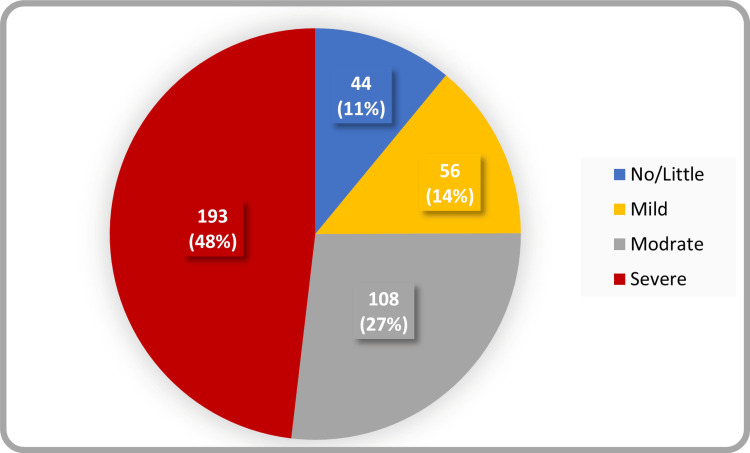
Severity of postmenopausal symptoms among surveyed women in the Qassim region, Saudi Arabia (n=401)

The response rate for this study was 95.2% (401/421). The menopausal transition had a substantial physical impact on the study population, with 193 women (48%) experiencing symptoms classified as severe. As indicated in Figure 1, this high symptom burden represented the predominant trend, followed by 108 (27%) participants reporting moderate discomfort and 56 (14%) experiencing mild symptoms. In contrast, only a small group of participants 44 (11%), reported few or no symptoms, indicating that most women in the Qassim region encounter significant physical or quality-of-life challenges during this stage. 

**Figure 2:**
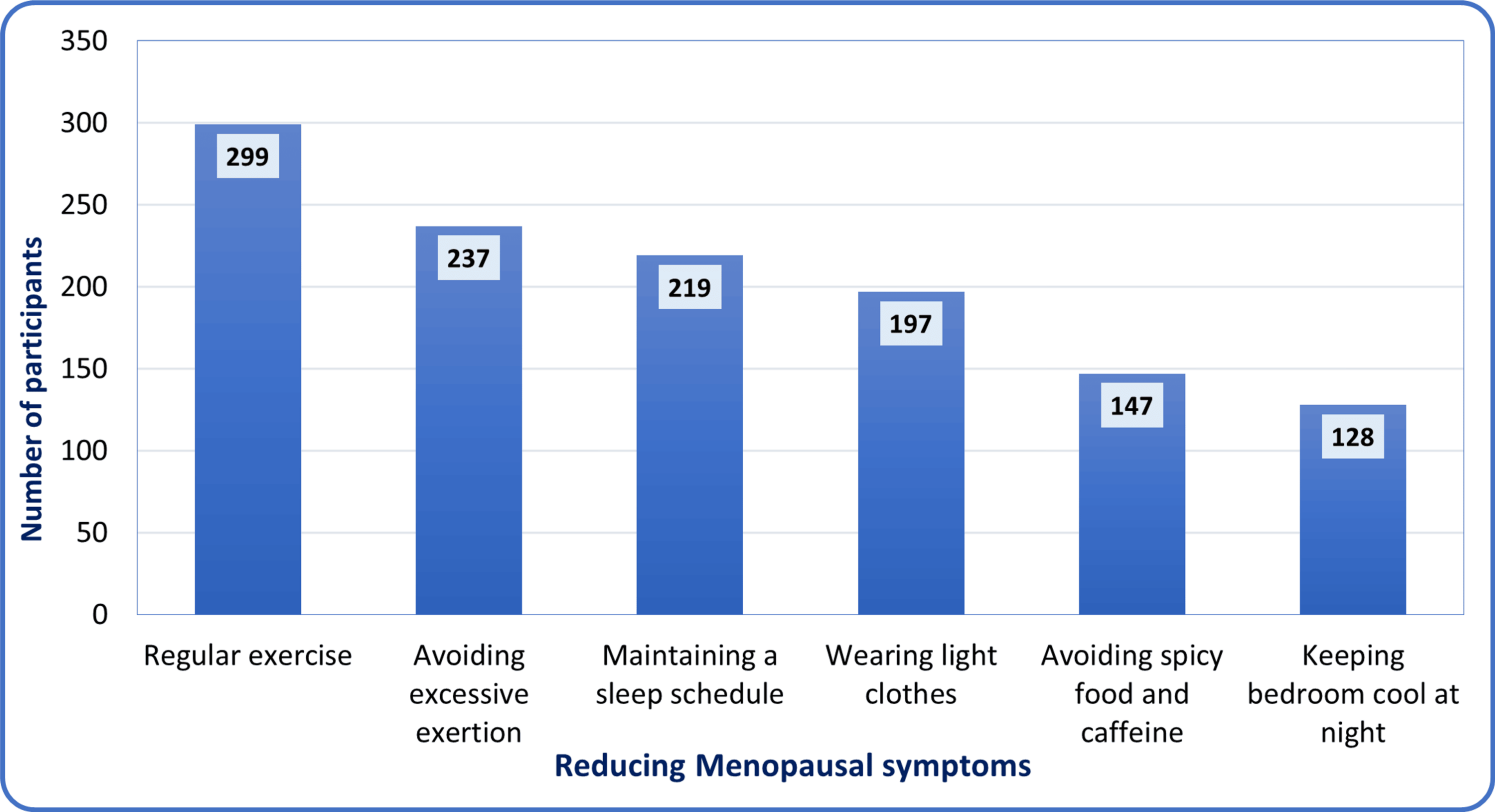
Adherence to useful guidelines for reducing menopausal symptoms among surveyed women in the Qassim region, Saudi Arabia (n=401)

Regarding the management of menopausal symptoms, participants showed a strong preference for non-pharmacological lifestyle changes. As shown in Figure 2, physical activity was the most common self-care method, with nearly three-quarters of the women,299 (74.6%), recommending regular exercise. In addition to physical activity, most respondents favored conservative adjustments to their daily routines, such as avoiding excessive physical exertion, 237 (59.1%), and keeping a regular sleep schedule, 219 (54.6%). Environmental and dietary modifications were also important, though less frequently used than behavioral changes. About half of the participants, 197 (49.1%), found relief through practical measures like wearing light clothing, while around a third adopted dietary restrictions, 147 (36.7%), or environmental cooling techniques at night, 128 (31.9%). Overall, these results suggest that women in the Qassim region prefer accessible, habit-based strategies to reduce their symptoms.

**Figure 3:**
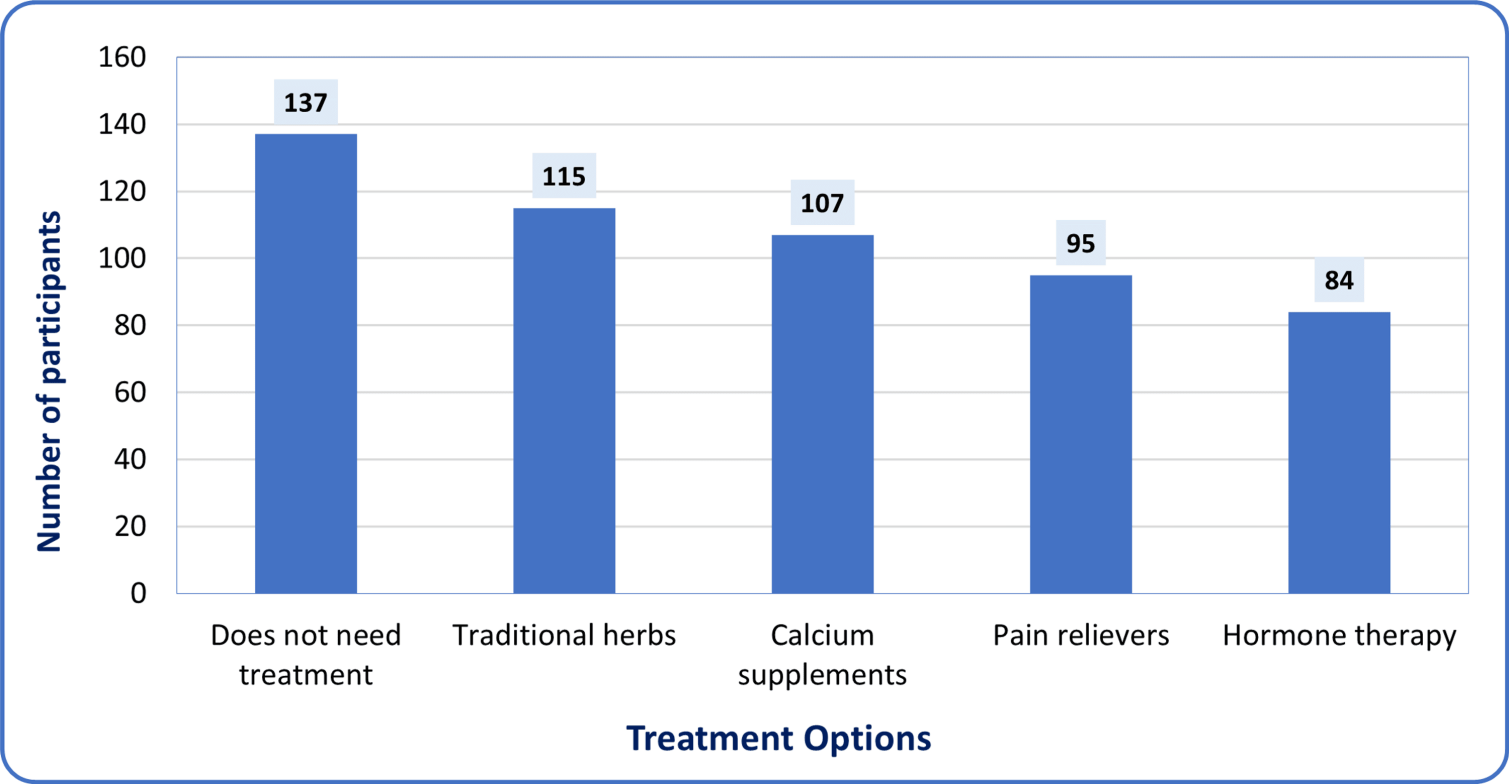
Most common treatment for reducing menopausal symptoms according to Saudi women (n=401)

When looking at preferences for clinical and alternative treatments, a clear trend appeared in how participants viewed the need for treatment. As shown in Figure 3, most women - 137 (34.2%) - felt that menopause does not require formal intervention. Among those who considered treatment, non-pharmaceutical and supplemental options were more popular than specialized medical therapies. Traditional herbal remedies were chosen most often, with 115 women (28.7%) preferring them. Calcium supplements were next, used by 107 (26.7%), followed by general pain relievers, taken by 95 (23.7%). Hormone therapy (HT), which is the standard clinical treatment for symptom relief, was the least preferred and chosen by only 84 (20.9%) participants. These choices suggest that many women are cautious or skeptical about hormonal treatments and prefer to manage symptoms themselves or use natural alternatives. 

**Figure 4:**
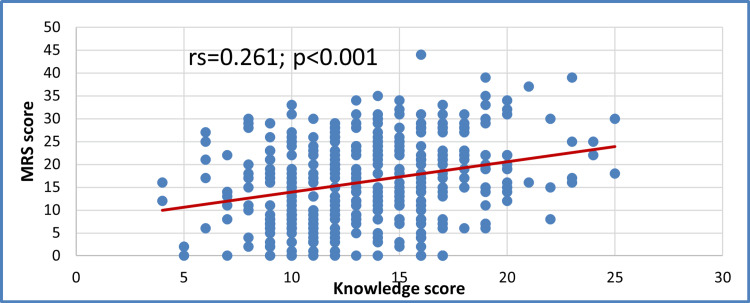
Correlation between MRS and knowledge scores Spearman’s rank correlation coefficient (rs = 0.261) indicates a significant positive correlation (p < 0.01) between menopausal knowledge scores and  MRS scores (n = 401) MRS: Menopause Rating Scale

The study also examined the relationship between a woman's knowledge of menopause and her perception of her physical condition. As shown in Figure 4, a clear and significant positive correlation (rs = 0.261, p < 0.001) was found between knowledge levels and the MRS scores. This indicates that as a woman’s understanding of the menopausal transition improves, she reports more symptoms. This moderate link may suggest that better-informed women are more aware of their health and more likely to notice and describe the subtle physiological and psychological changes they experience, rather than dismissing them as unrelated issues.

The sociodemographic profile of the 401 women surveyed were mostly in the mid-to-late stages of menopause, with an average age of 48.8 years (±7.68). Table 1 shows that the largest group was aged 46-55, making up 167 women (41.6%). Most participants had stable social backgrounds: 330 (82.3%) were married, 264 (65.8%) lived in urban areas, and 207 (51.6%) had three to five children. This group stood out for having both high levels of education and traditional domestic roles. Over half 236 (58.9%) had university degrees, and almost all 397 (99%) described themselves as housewives. Economically, most fell into the lower-to-middle income range, with 138 (34.4%) earning less than Saudi Arabian Riyal (SAR) 5,000 per month and 49 (12.2%) earning more than SAR 15,000. This mix of education and homemaking shapes how menopause is viewed and managed in the Qassim region. 

**Table 1:** Sociodemographic characteristics of surveyed women in Qassim region, Saudi Arabia (n=401) Data are presented as Frequency (n) and Percentage (%). SAR: Saudi Arabian riyal

Study variables	Frequency (n)	Percentage (%)
Age group		
< 35 years	13	3.2%
36–45 years	135	33.7%
46–55 years	167	41.6%
> 55 years	86	21.4%
Marital status		
Single	4	1%
Married	330	82.3%
Divorced	42	10.5%
Widowed	25	6.2%
Place of residence		
City	264	65.8%
Governorate	113	28.2%
Peripheral village	24	6%
Educational level		
No formal education	18	4.5%
Primary school	40	10%
Secondary school	91	22.7%
University	236	58.9%
Postgraduate studies	16	4%
Employment status		
Housewife	397	99%
Employed	3	0.7%
Unemployed	1	0.3%
Monthly income (SAR)		
< 5,000	138	34.4%
5,000–10,000	122	30.4%
10,001–15,000	92	22.9%
> 15,000	49	12.2%
Number of children		
< 3 children	59	14.7%
3–5 children	207	51.6%
> 5 children	135	33.7%

Women in the Qassim region often reported a high prevalence of physical and somatic symptoms. Table 2 shows that joint and muscle discomfort is the most common and severe problem, with the highest average severity score of 1.96 (±1.39). Many women also experience physical and mental exhaustion (1.70 ±1.34). Along with typical vasomotor symptoms like hot flashes and sweating (1.72 ±1.34), these issues are the main challenges for participants. Sleep problems (1.61 ±1.30) are also common, showing that physical health and vasomotor symptoms are the most affected areas. Other reported symptoms included depressed mood (1.58 ±1.28), vaginal dryness (1.45 ±1.39), and equal scores for both tension (1.35 ±1.22) and anxiety (1.35 ±1.27). In contrast, other symptom categories were reported less often and with lower intensity. Cardiovascular discomfort is less severe (1.03 ±1.10), and problems with sexual health (1.24 ±1.22) or bladder function (1.26 ±1.29) are among the least reported. The average MRS score is 16.3 (±9.22), suggesting that although some health aspects are stable, women in this region generally experience a moderate yet varied set of symptoms that affect their daily physical well-being. 

**Table 2:** : Self-reported prevalence and severity of menopausal symptoms among surveyed women in the Qassim region, Saudi Arabia (n=401) MRS categories used with permission from ZEG Berlin GmbH. Descriptive prompts were removed to comply with open-access copyright restrictions. For detailed methodology and validation of the scale, please refer to the original literature published by BioMed Central [11]. MRS: Menopause Rating Scale

MRS Domains	Mean	SD
Hot flashes, sweating	1.72	1.34
Heart discomfort	1.03	1.10
Sleep problems	1.61	1.30
Depressed mood	1.58	1.28
Tension	1.35	1.22
Anxiety	1.35	1.27
Physical and mental exhaustion	1.70	1.34
Sexual problems	1.24	1.22
Bladder problems	1.26	1.29
Vaginal dryness	1.45	1.39
Joint and muscle discomfort	1.96	1.39
Total MRS score	16.3	9.22

The assessment of menopausal literacy among participants revealed a significant gap between understanding basic biological facts and recognizing more complex symptoms. As shown in Table 3, there was high agreement regarding the nature of menopause, with most correctly identifying it as a natural phase of life, 368 (91.8%), and acknowledging the end of fertility, 349 (87%). Most women also understood that this transition signifies the permanent end of menstruation, 312 (77.8%), and maintained a positive outlook on sexual health during this period. However, a closer look at the data uncovers notable knowledge gaps and cultural misconceptions. Less than half of the participants, 175 (43.6%), could accurately define the technical timeframe for postmenopause, and a similarly low number, 186 (46.4%), recognized the clinical importance of consulting a doctor during this stage. Furthermore, deeply rooted social perceptions were evident: only a small fraction of participants, 26 (6.5%), disagreed with the idea that menopause affects femininity, and even fewer, 22 (5.5%), challenged the notion of societal devaluation in postmenopausal years. This inconsistency extended to symptom recognition. While common symptoms like hot flashes, 253 (63.1%), and mood swings, 230 (57.4%), were well known, cognitive and physical issues remained largely unnoticed. Only 88 (21.9%) identified concentration problems as a symptom, and even fewer recognized signs such as chills or urinary changes. Overall, the average knowledge score of 13.5 (±3.81) indicates a population knowledgeable about the basic "what" of menopause but lacking a full understanding of the "how" and "why" related to long-term management and diverse symptom presentation.

**Table 3:** Assessment of the knowledge toward postmenopausal symptoms and management (n=401) Data are presented as Frequency (n) and Percentage (%). Results regarding "Level of Knowledge" are categorized based on the total knowledge score (Mean ± SD: 13.5 ± 3.81).

Knowledge items	Yes N (%)	No N (%)	I don't know/Maybe
Condition related to menopause post-menopause (menstruation stopped for more than one year)	175 (43.6%)	177 (44.1%)	49 (12.2%)
Menopause is the permanent cessation of menstruation	312 (77.8%)	63 (15.7%)	26 (6.5%)
Menopause means losing the ability to conceive naturally	349 (87.0%)	27 (6.7%)	25 (6.2%)
Menopause is a natural process	368 (91.8%)	21 (5.2%)	12 (3.0%)
Is menopause a natural aging process?	191 (47.6%)	144 (35.9%)	66 (16.5%)
Does a woman gain more value in society as a mature person after menopause?	202 (50.4%)	109 (27.2%)	90 (22.4%)
Does menopause mean there will be no menstrual bleeding?	203 (50.6%)	134 (33.4%)	64 (16.0%)
Is it possible to engage in sexual activities during menopause?	312 (77.8%)	32 (8.0%)	57 (14.2%)
Does a woman's psyche change after menopause?	217 (54.1%)	76 (19.0%)	108 (26.9%)
Should a woman visit a doctor when she reaches menopause?	186 (46.4%)	125 (31.2%)	90 (22.4%)
Does a woman lose her femininity after menopause?	26 (6.5%)	328 (81.8%)	47 (11.7%)
Does a woman lose her value in society after menopause?	22 (5.5%)	358 (89.3%)	21 (5.2%)
Symptoms of menopause	Yes N (%)	No N (%)	—
Hot flashes, sweating (episodes of sweating)	253 (63.1%)	148 (36.9%)	—
Mood changes	230 (57.4%)	171 (42.6%)	—
Irregular menstrual cycle	220 (54.9%)	181 (45.1%)	—
Vaginal dryness	202 (50.4%)	199 (49.6%)	—
Joint pains	197 (49.1%)	204 (50.9%)	—
Sleep disturbance	172 (42.9%)	229 (57.1%)	—
Headache	144 (35.9%)	257 (64.1%)	—
Night sweats	139 (34.7%)	262 (65.3%)	—
Emotional changes	134 (33.4%)	267 (66.6%)	—
Weight gain	128 (31.9%)	273 (68.1%)	—
Hair thinning and dry skin	127 (31.7%)	274 (68.3%)	—
Problems with concentration and learning	88 (21.9%)	313 (78.1%)	—
Chills	63 (15.7%)	338 (84.3%)	—
Early urination	57 (14.2%)	344 (85.8%)	—
Total knowledge score (mean ± SD)	13.5 ± 3.81	—	—
Level of knowledge	N (%)	—	—
Poor	127 (31.7%)	—	—
Moderate	154 (38.4%)	—	—
Good	120 (29.9%)	—	—

Participants showed a wide range of knowledge about menopause, with several sociodemographic factors contributing significantly to this variation. Table 4 reveals that age and financial stability were the primary factors linked to higher menopausal literacy. Women aged ≥45 years achieved higher knowledge scores (13.9 ± 3.74) compared to those <45 years (12.8 ± 3.81), a difference that was statistically significant (p = 0.002). Similarly, women earning ≥10,000 SAR per month scored significantly higher (14.3 ± 3.79) than those with lower incomes (13.1 ± 3.76; p = 0.009). Family size also played a role; women with >5 children had the highest average scores (14.2 ± 3.462), suggesting that personal experience and interactions within larger families may enhance menopausal understanding (p = 0.041).In contrast, other factors typically associated with awareness had negligible effects. Marital status (Unmarried: 14.2±4.38 vs. Married: 13.4 ± 3.66; p=0.140) and place of residence (City: 13.3 ± 3.71, Governorate: 13.9 ± 4.00, and Peripheral village: 13.8 ± 3.83) showed no significant differences (p = 0.265). Furthermore, formal education did not lead to substantial variation in scores; while women with university or higher education had higher averages (13.7 ± 3.96) than those with secondary education or below (13.2 ± 3.53), the difference was not statistically significant (p = 0.311). This suggests that in the Qassim region, menopausal knowledge may be disseminated more through cultural and family networks than formal schooling. Finally, a moderate positive link between knowledge and symptom reporting suggests that higher literacy may increase awareness of physical and psychological changes.

**Table 4:** Differences in knowledge score in relation to the sociodemographic characteristics of the Saudi women (n=401) Results are presented as Mean ± SD. Statistical significance was determined using the following tests § P-value has been calculated using Mann-Whitney U-test.(Z-statistic); ‡ P-value has been calculated using Kruskal Wallis H-test; ** Significant at p<0.05 level; *26: number of questions in the questionnaire assessing participants' knowledge SAR: Saudi Arabian riyal

Factor	Knowledge Score (26*) Mean ± SD	Z/H-tests	P-value §
Age group : < 45 years	12.8 ± 3.81	3.046	0.002**
Age group: ≥ 45 years	13.9 ± 3.74	-	-
Marital status : Unmarried	14.2 ± 4.38	1.475	0.140
Marital status: Married	13.4 ± 3.66	-	-
Place of residence ‡: City	13.3 ± 3.71	2.653	0.265
Place of residence: Governorate	13.9 ± 4.00	-	-
Place of residence: Peripheral village	13.8 ± 3.83	-	-
Educational level: Secondary or below	13.2 ± 3.53	1.014	0.311
Educational level: University or higher	13.7 ± 3.96	-	-
Monthly income (SAR): < 10,000	13.1 ± 3.76	2.609	0.009**
Monthly income (SAR): ≥ 10,000	14.3 ± 3.79	-	-
Number of children ‡: < 3 children	12.9 ± 4.08	6.380	0.041**
Number of children: 3–5 children	13.3 ± 3.79	-	-
Number of children: > 5 children	14.2 ± 3.462	-	-

## Discussion

This study explores how well Saudi women in the Qassim region understand postmenopausal symptoms and their attitudes toward managing them. The findings are important because they highlight significant gaps in awareness and knowledge among these women. The findings may reveal unique cultural and regional factors that shape how women experience and respond to menopause. The findings can also help create tailored health education programs and guide healthcare providers in offering culturally sensitive support.

Regarding knowledge of postmenopausal symptoms and management, findings suggest a moderate level of understanding among women (mean score: 13.5 out of 26 points). Notably, only 29.9% demonstrated a solid grasp of the subject. This finding closely mirrored a previous study, which reported that 30.2% of women were well aware of perimenopausal symptoms [16]. However, our results differed from previous findings, which reported lower levels of knowledge among primary care patients. This discrepancy may stem from variations in educational backgrounds or differences between urban and rural areas [17]. The lack of awareness regarding postmenopausal symptoms and their management is evident in our findings. To close the knowledge gap on menopause, it is necessary to develop health education programs that account for cultural contexts. It is also essential to enhance the training for healthcare providers so they can have open and proactive discussions about menopause with the women they treat. Offering accessible resources, like community workshops and online platforms, can make a big difference. These initiatives should aim to make conversations about menopause more commonplace, debunk the myths, and empower women by giving them accurate information and practical advice for handling their symptoms effectively.

Sociodemographic factors played a significant role in shaping knowledge. Women aged >45 years, those with higher income, and those with more children had significantly better knowledge scores. Potential reasons for these effects may include the following: Higher income often enables greater access to healthcare services, educational resources, and health-related information, allowing these women to consult professionals and participate in health programs. Additionally, having more children typically increases interactions with healthcare providers over the years, which can lead to more opportunities to learn about women's health issues, including menopause. Life experiences gained through raising children and managing family health may also foster a proactive attitude towards personal well-being, encouraging these women to seek information and adopt effective strategies to manage postmenopausal symptoms.

This trend is consistent with previous studies, which found that both age and the number of children a woman has are positively associated with her understanding of menopause. This could be attributed to personal experiences and greater access to health information over time [7,15]. Interestingly, although other studies have identified education level as a strong factor in menopausal knowledge [15,16], our study did not find a significant association. However, we did notice that women with a university education tended to have slightly higher scores. This could indicate a ceiling effect within our population, or it may reflect the impact of informal sources of knowledge, such as social media, which has been reported as the most commonly used information channel [18].

In our study, 91.8% of participants correctly identified menopause as a natural process, and 87.0% acknowledged the loss of fertility, which is notably higher than the 56% general knowledge level reported by a previous national study of Saudi women [18]. This discrepancy may reflect regional differences in access to health education or sampling variation, as our study focused on a more health-literate cohort. However, previous findings have shown that only 40% of women had accurate knowledge of postmenopause [7]. Our study revealed that only 43.6% correctly defined postmenopause as cessation of menstruation for over a year, indicating a consistent national gap in understanding this clinical definition.

Symptom recognition in our cohort was moderate for vasomotor and psychological symptoms - hot flashes and mood changes - but poor for cognitive and somatic symptoms such as chills and early urination. These findings align with previous reports indicating that while hot flashes and mood swings are commonly recognized, cognitive and urogenital symptoms are often overlooked [19]. Similarly, previous findings from Al-Ahsa found that women who engaged in physical activity and self-care were more likely to recognize and manage symptoms, suggesting that lifestyle and health-seeking behaviors may influence symptom awareness [20].

Our findings also shed light on some common misunderstandings within society. For instance, only 6.5% of people rejected the idea that women lose their femininity after menopause, and just 5.5% denied that there is a societal devaluation of women during this stage. These attitudes are similar to those previously reported, indicating that social support and cultural narratives heavily influence how women view menopause in Taif [21]. These beliefs often originate from deeply rooted gender norms and a lack of open discussion about aging and female sexuality, a sentiment that is supported by wider qualitative studies conducted across Saudi Arabia [22].

Experiencing postmenopausal symptoms often drives women to seek out information and support, which can help them better understand menopause and how to manage it effectively. In our study, we found that joint and muscle discomfort were the most commonly reported symptoms among the women we surveyed, followed by issues with vasomotor symptoms and fatigue. This corroborates previous findings indicating that joint pain and exhaustion were the leading symptoms among Saudi women visiting primary care clinics in Riyadh [19]. Similarly, earlier findings reported that physical and mental exhaustion (80.3%) and joint discomfort (79.2%) were prevalent in Taif [21]. Interestingly, our sample exhibited a greater prevalence of severe symptoms 49.1% compared with prior reports, which documented 25.2% of women experienced severe joint pain and 20.2% reported severe exhaustion [17]. These differences might stem from variations in healthcare access, cultural perspectives on symptoms, or the ways symptoms are reported. Looking at international data, these disparities become even more apparent. Studies have reported that 32 to 46% of women in Europe and the United States experienced moderate to severe vasomotor symptoms, along with higher rates of clinical intervention [23]. Our findings suggest that women in Qassim fall on the higher end of the global spectrum regarding symptom severity but show lower engagement in seeking clinical care.

Traditional herbs (28.7%) and calcium supplements (26.7%) were the most commonly used treatments, while 34.2% of participants believed that menopausal symptoms did not require treatment. This mirrors the findings of a prior study, which reported low uptake of HT and a preference for self-management among Saudi women [7]. Contrary to these reports, symptomatic women in the United States who sought medical care were prevalent, with 34% using HT [24]. The reluctance to seek medical support in our cohort may be influenced by cultural norms, limited awareness, or perceived stigma, as also observed among ethnic minority populations in the UK [25].

The positive correlation between MRS scores and knowledge (rs = 0.261, p < 0.001) suggests that more symptomatic women may be more motivated to seek information, a trend also observed in studies from Korea and Turkey [26,27]. This reinforces the need for proactive education before symptom onset to empower women with anticipatory guidance rather than reactive learning.

Strengths and Limitations

The primary strength of this study is its large sample size (401), which provided sufficient statistical power to detect significant associations between sociodemographic factors and menopausal knowledge. By exceeding the initial power calculation, the results reliably reflect the perceptions of women in the primary healthcare setting. However, several limitations need to be acknowledged. First, the cross-sectional design prevents establishing causal relationships between knowledge levels and symptom severity; longitudinal studies are necessary to observe these trends over time. Second, the study used a non-probability convenience sampling method, which may limit the generalizability of the results to the broader population. Additionally, the geographic focus was limited to the Qassim region, so the findings may not fully represent women in other parts of Saudi Arabia with different socioeconomic or healthcare environments. Third, relying on self-reported data introduces potential recall or social desirability bias, especially on sensitive topics like sexual health. Fourth, although the MRS is a globally validated tool, qualitative approaches could provide deeper cultural insights into symptom experiences. Lastly, the high percentage of housewives in our sample 397 (99%) might influence the outcomes, as employment status often affects health-seeking behaviors and access to health information.

## Conclusions

In conclusion, this study reveals a high prevalence of menopausal symptoms among women in the Qassim region, contrasted by a moderate level of knowledge regarding their management. Our analysis identified significant sociodemographic factors that influence this awareness, highlighting a critical gap between symptom recognition and professional medical consultation. These findings suggest that current healthcare strategies must transition toward culturally tailored educational programs to improve health-seeking behaviors and evidence-based management for menopausal women in Saudi Arabia.

## Appendices

Appendix 1

Questionnaire

You are invited to participate in our study that aims to assess the knowledge and attitudes of Saudi women who attended PHCCs in Buraidah city, Qassim region, Saudi Arabia, regarding postmenopausal symptoms and their management. The purpose is to evaluate awareness levels and perceptions related to these topics. All information collected will remain confidential and will only be used for research purposes. Your identity will not be disclosed in any publications or presentations resulting from this study. Do you agree to participate voluntarily?

a. Yes

b. No

**Table 5:** Section One - Demographic Data Credits: Renad Almugbel, Rand Albahli, and Amel Abdalrahim Sulaiman SAR: Saudi Arabian riyal

Field	Response Options
Age	( ) Years
Marital Status	Single / Married / Divorced / Widowed
Place of Residence	City / Governorate / Peripheral Village
Education Level	No formal education / Primary / Secondary / University / Postgraduate
Employment Status	Employed / Unemployed / Retired / Housewife / Freelance / Student
*Income Level (SAR)	< 5,000 / 5,000–10,000 / 11,000–15,000 / > 15,000
Number of Children	( )
Menopausal Status	Pre-menopause / post-menopause / I don’t know

**Table 6:** Section Two - General Knowledge about Menopause and MRS Credits: Renad Almugbel, Rand Albahli, and Amel Abdalrahim Sulaiman MRS: Menopause Rating Scale

Question	Yes	No	Maybe / I don't know
Is menopause the permanent cessation of menstruation?	□	□	□
Does menopause mean losing the ability to conceive naturally?	□	□	□
Is menopause a natural aging process?	□	□	□
Does a woman gain more value in society as she matures?	□	□	□
Is it possible to engage in sexual activities during menopause?	□	□	□
Does a woman’s psyche/mood change after menopause?	□	□	□
Should a woman visit a doctor when she reaches menopause?	□	□	□
Does a woman lose her femininity after menopause?	□	□	□
Does a woman lose her value in society after menopause?	□	□	□
Is menopause a frightening event for you?	□	□	□

**Table 7:** Lifestyle and Treatment Guidelines for Reducing Symptoms: You can select more than one option. Credits: Renad Almugbel, Rand Albahli, and Amel Abdalrahim Sulaiman

Category	Option / Recommendation	Selection
Guidelines for Reducing Symptoms	Wearing light clothes	□
	Avoiding excessive exertion	□
	Regular exercise	□
	Avoiding spicy food and caffeine	□
	Maintaining a sleep schedule	□
	Keeping the bedroom cool at night	□
Preferred Treatment Method	Hormone therapy	□
	Traditional herbs	□
	Calcium supplements	□
	Pain relievers	□
	Does not need treatment	□
